# Visualisation of Chemical Shielding Tensors (VIST) to Elucidate Aromaticity and Antiaromaticity**

**DOI:** 10.1002/ejoc.202100352

**Published:** 2021-05-05

**Authors:** Felix Plasser, Florian Glöcklhofer

**Affiliations:** ^1^ Department of Chemistry Loughborough University Loughborough LE11 3TU United Kingdom; ^2^ Department of Chemistry and Centre for Processable Electronics Imperial College London Molecular Sciences Research Hub London W12 0BZ United Kingdom

**Keywords:** Aromaticity, Antiaromaticity, Chemical shift, Hydrocarbons, *π*-Conjugated systems

## Abstract

Aromaticity is a central concept in chemistry, pervading areas from biochemistry to materials science. Recently, chemists also started to exploit intricate phenomena such as the interplay of local and global (anti)aromaticity or aromaticity in non‐planar systems and three dimensions. These phenomena pose new challenges in terms of our fundamental understanding and the practical visualisation of aromaticity. To overcome these challenges, a method for the visualisation of chemical shielding tensors (VIST) is developed here that allows for a 3D visualisation with quantitative information about the local variations and anisotropy of the chemical shielding. After exemplifying the method in different planar hydrocarbons, we study two non‐planar macrocycles to show the unique benefits of the VIST method for molecules with competing *π*‐conjugated systems and conclude with a norcorrole dimer showing clear evidence of through‐space aromaticity. We believe that the VIST method will be a highly valuable addition to the computational toolbox.

## Graphical abstract

A method for the visualisation of chemical shielding tensors (VIST) is developed, which provides a graphical representation of aromaticity and antiaromaticity, their local variations, and anisotropy. The method provides unique insight into fascinating phenomena such as the interplay of aromaticity and antiaromaticity in macrocycles with competing *π*‐conjugated systems.

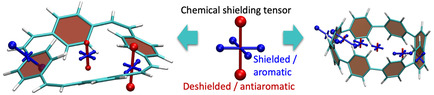

## Introduction

The concept of aromaticity has intrigued chemists for over 200 years^[1]^ and is a fundamental ingredient in our understanding of the properties and reactivity of molecules. Aromatic molecules play central roles in many areas of chemistry, such as organic chemistry, biochemistry, photochemistry, and molecular materials science. More recently, chemists also started to consider local aromatic effects and antiaromaticity in the design of organic materials offering promising applications based on fascinating chemistry. The field of singlet fission,^[2]^ as one prominent example, has been invigorated by both ideas, and the modulation of local aromaticity via the insertion of heteroatoms[^3^, ^4^] and the interplay of ground‐state antiaromaticity with triplet excited‐state aromaticity[^5^, ^6^, ^7^] have led to a new push in the quest for molecules with the desired energies of their singlet and triplet excited states. Moreover, the application of Clar's sextet theory^[8]^ to control local aromaticity provides a powerful way of tuning biradical character, thus, opening the route to a range of optoelectronic applications.[^9^, ^10^, ^11^, ^12^]

A new and exciting frontier is opened in terms of macrocycles and larger *π*‐conjugated systems, in which local and global (anti)aromaticity can both play a role. Here, (anti)aromaticity is being studied in systems as diverse as nanographenes,[^13^, ^14^] porphyrin nanorings,[^15^, ^16^] carbon nanobelts,^[17]^ cyclocarbon,^[18]^ cycloparaphenylenes,[^19^, ^20^, ^21^] cycloparaphenylmethine,^[22]^ paracyclophanetetraene,[^23^, ^24^] norcorrole[^25^, ^26^] and other porphyrinoids.[^27^, ^28^] Several of these systems are interesting due to their remarkable capacity to stabilise multiply charged ions^[20]^ making them promising candidates for organic battery electrodes.[^24^, ^29^, ^30^] Excited‐state (triplet) aromaticity[^31^, ^32^] and Möbius aromaticity are also being investigated,[^33^, ^34^, ^35^, ^36^] as well as three‐dimensional aromaticity in stacked systems,^[37]^ cyclophanes,^[38]^ *π*‐conjugated cages,^[39]^ and borane cages.^[40]^ Finally, homoaromaticity provides yet another fascinating field of non‐standard aromaticity[^41^, ^42^, ^43^, ^44^] with potential applications in mechanoresponsive materials.^[43]^

Considering the ubiquity of aromaticity in chemistry and its diverse appearances, there has been a strong push toward the development of methods to visualise and quantify aromaticity in different instances, many of which are related to the characteristic signals of (anti)aromatic systems in nuclear magnetic resonance (NMR) spectroscopy. Popular approaches rely on current densities, such as the anisotropy of the induced current density (ACID),^[45]^ the gauge including magnetically induced current (GIMIC),[^46^, ^47^] or other current density maps.^[48]^ Alternatively, it has been suggested to visualise induced magnetic fields.^[49]^ A prominent quantitative measure for aromaticity is provided by the nucleus‐independent chemical shifts (NICS)[^50^, ^51^] corresponding to an NMR experiment performed at a virtual nucleus at the centre of an aromatic ring. NICS values can be visualised after computing them on a grid around the molecule of interest[^52^, ^53^, ^54^, ^55^] and several recent applications present 1D scans, 2D contour plots, or even 3D isosurfaces of the NICS values, e. g. to visualize local aromatic and antiaromatic parts of larger molecules,[^6^, ^25^, ^38^, ^56^] to represent Clar sextets in condensed hydrocarbons,^[57]^ or to study interactions in excimers.^[58]^ These visualisations are almost exclusively based on isotropic NICS values. However, new challenges come into play in macrocycles and multi‐ring systems where several ring currents in different planes interact, possibly resulting in strong magnetic anisotropy. To overcome this problem, it would be greatly beneficial to have a method for the visualisation of the full underlying shielding tensor.

**Figure 1:**
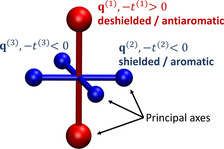
Visualisation of the chemical shielding tensor (VIST) via its three principal axes ${\vec{q}}^{\left(1\right)}$ , ${\vec{q}}^{\left(2\right)}$ , ${\vec{q}}^{\left(3\right)}$ and the associated eigenvalues *t* ^(1)^, *t* ^(2)^, *t* ^(3)^.

The purpose of this work is to develop such a method, denoted VIST (visualisation of chemical shielding tensors). VIST will allow us to visualise local variations in aromaticity and antiaromaticity in the context of the molecular structure while also providing insight into the anisotropy of the chemical shielding. Briefly spoken, the method proceeds by computing the chemical shielding tensor at a given point in space, computing its principal axes via a diagonalisation, and showing them as a local coordinate system. The method is sketched in Figure 1. In this example, there is one strong deshielded (antiaromatic) component shown in red and two weaker shielded (aromatic) components shown in blue. Any one of these tensor components relates to the ring currents in a plane perpendicular to it.

Within this work, we first discuss the underlying theory and working equations of the VIST method. Subsequently, we apply the method to benzene, phenanthrene, and cyclobuta[l]phenanthrene to highlight the interplay of local aromaticity and antiaromaticity in these molecules and their variations with the electronic spin state. The full power of the VIST method is illustrated in three non‐planar macrocyclic systems – paracyclophanetetraene (PCT), [8]cycloparaphenylene ([8]CPP), and a norcorrole dimer – to study the interplay of local aromaticity and antiaromaticity in these molecules and their variations for electronic states of different charge.

## Computational Methods

### Definition of the chemical shielding tensors

The chemical shielding, which underlies NMR spectroscopy, is generally defined as the mixed second derivative of the energy with respect to an external magnetic field *B*_*β*_ and the nuclear magnetic moment *μ*_*γ*_[^54^, ^59^, ^60^]

(1) $$\sigma_{\gamma \beta }=\frac{\partial^{2}E}{\partial B_{\beta }\partial \mu_{\gamma }}$$

where *β* and *γ* are two Cartesian coordinates (x, y, z). Usually, the shielding is given as a sum of a diamagnetic $\sigma_{\gamma \beta }^{dia}$ and paramagnetic $\sigma_{\gamma \beta }^{para}$ term:

(2) $$\sigma_{\gamma \beta }=\sigma_{\gamma \beta }^{dia}+\sigma_{\gamma \beta }^{para}$$

The properties of these terms have been discussed in great detail[^54^, ^60^, ^61^] and we shall only mention them briefly here. Assuming that the gauge origin lies at the point probed, we can write the diagonal z‐component of the diamagnetic term as

(3) $$\sigma_{zz}^{dia}=\frac{\alpha^{2}}{2}\int \frac{{x^{2}+y}^{2}}{r^{3}}\rho \left(\;\vec{r}\right)d\vec{r}=\frac{\alpha^{2}}{2}\int \frac{\sin^{2}\theta }{r}\rho \left(\;\vec{r}\right)d\vec{r}\geq 0$$

where *α* is the fine structure constant, $\rho \left(\vec{r}\right)$ is the electron density at position $\vec{r}$ , and *θ* is the angle from the z‐axis. Analogous equations hold for $\sigma_{xx}^{dia}$ and $\sigma_{yy}^{dia}$ . This term, related to the Lamb formula, represents the textbook view of NMR spectroscopy – the chemical shielding increases with the electron density $\rho \left(\vec{r}\right)$ around the nucleus of interest.[^61^, ^62^] Closer inspection of Eq. (3) shows that the chemical shielding in any given direction is determined by the electron distribution in the plane perpendicular to it. Thus, ring currents in any given plane are represented by a pronounced tensor component perpendicular to that plane.

The paramagnetic term is usually given in a perturbative expansion[^54^, ^63^] and, viewing again only its diagonal z‐component at the gauge origin, we can write

(4) $$\sigma_{zz}^{para}=-\alpha^{2}\sum_{I\neq 0}\frac{\langle \Psi_{0}|{\hat{L}}_{z}/r^{3}|\Psi_{I}\rangle \langle \Psi_{I}|{\hat{L}}_{z}\left|\Psi_{0}\right\rangle }{E_{I}-E_{0}}$$

where ${\hat{L}}_{z}$ denotes the angular momentum operator, Ψ_0_ and Ψ_*I*_ are the ground and excited‐state wavefunctions, and *E*_0_ and *E_I_* are their energies. $\sigma_{zz}^{para}$ is generally a negative/deshielding term opposing the diamagnetic term.^[61]^

It is the idea behind the nucleus independent chemical shift (NICS) method^[51]^ to compute the shielding not only at the nuclei, as relevant to NMR spectroscopy, but also at other points in space to gain insight into (anti)aromaticity. The NICS at any given point in space is defined as the negative of the isotropically averaged chemical shielding, i. e.

(5) $$NICS=-\sigma_{iso}=-\frac{1}{3}\left(\sigma_{xx}+\sigma_{yy}+\sigma_{zz}\right).$$

Note that the chemical shielding and NICS are thus equivalent except for the sign and we shall use both terms interchangeably. It is known that NICS values computed at the centre of an aromatic ring are negative, whereas they are positive for an antiaromatic ring.^[51]^ Considering Eq. (3) and Eq. (4), we can rephrase this in the sense that for an aromatic system diamagnetic shielding dominates, whereas for an antiaromatic system paramagnetic deshielding dominates. The diamagnetic shielding only depends on the distribution of the electrons in space and is, thus, present for aromatic and antiaromatic systems alike. Paramagnetic deshielding, on the other hand, has a more involved expression relying on the existence of low‐energy excited states accessible through rotational transitions (cf. Refs 64,65) and becomes large in magnitude only for antiaromatic systems. In the context of the above discussion it should be noted that only the total shielding is invariant to the gauge origin whereas the division between diamagnetic and paramagnetic shielding depends on the gauge origin chosen.^[60]^ The presented discussion, therefore, represents only one possible interpretation of the underlying physics.

### Visualisation of shielding tensors (VIST)

Following Eq. (1), it is seen that the chemical shielding is given as a non‐symmetric 3×3 tensor containing 9 independent values. Whereas scalar‐valued functions can be represented in 3D space *via* isosurfaces and vector‐valued functions *via* arrows, it is necessary to construct a more involved representation for a tensor‐valued function. Here, we suggest doing so by constructing the principal axes of the chemical shielding tensor as its eigenvectors in analogy to the principal axes of the moment of inertia. The eigenvectors ${\vec{q}}^{\left(1\right)}$ , ${\vec{q}}^{\left(2\right)}$ , ${\vec{q}}^{\left(3\right)}$ and eigenvalues *t* ^(1)^, *t* ^(2)^, *t* ^(3)^ are given as

(6) $$\sum_{\beta \in \left\{x,y,z\right\}}\sigma_{\gamma \beta }q_{\beta }^{\left(i\right)}=t^{\left(i\right)}q_{\gamma }^{\left(i\right)}\;\;\;i\in \left\{1,2,3\right\}$$

For visualisation, we construct a local coordinate system oriented according to the eigenvectors ${\vec{q}}^{\left(i\right)}$ , and visualise the three components as dumb‐bells whose size and length depend on the absolute value of the associated eigenvalue |*t* ^(*i*)^| and whose color depends on the sign (blue or red), see Figure 1. Through encoding the eigenvectors and eigenvalues in the representation, we are able to represent the full information given in the 3×3 tensor graphically. To compare these results to the NICS values, it is worth noting that in analogy to Eq. (5) the NICS value is a third of the sum of the three eigenvalues according to

(7) $$NICS=-\sigma_{iso}=-\frac{1}{3}\left(t^{\left(1\right)}+t^{\left(2\right)}+t^{\left(3\right)}\right).$$

Finally, we want to point out that the shielding tensor is in general represented by a non‐symmetric matrix, which gives rise to two technical points to consider: (i) the fact that the left and right eigenvectors are not the same and (ii) the occurrence of complex eigenvalues. Both points are discussed in Sec. S1 of the supporting information.

### Computational details

Chemical shielding tensors were computed in Gaussian 09^[66]^ using the PBE0 functional[^67^, ^68^] along with the def2‐SVP basis set^[69]^ using gauge‐including atomic orbitals^[59]^ and applying tight SCF convergence criteria. Calculations on the singlet (triplet) states were performed using restricted (unrestricted) density functional theory. Molecular geometries of benzene, phenanthrene, cyclobuta[l]phenanthrene, PCT, and [8]CPP were optimised at the PBE0/def2‐SVP level. Geometries were optimised for all different electronic states considered (singlet, triplet, dication, dianion). These individually optimised geometries were used as a basis to compute the shielding tensors for the different states. In the case of the stacked norcorrole dimer, the molecular geometry was taken from the crystal structure^[38]^ with removed bithiophene linkers. The same structure, but with one monomer removed, was used for the monomer calculation.

We have implemented the VIST method within the TheoDORE wavefunction analysis package.[^70^, ^71^, ^72^] A first version of the code has been released within TheoDORE 2.4. The visual molecular dynamics (VMD)^[73]^ program is used as a graphical backend for creating the tensor representations in connection with molecular structures and isosurfaces. The signed current density modulus^[46]^ plots were computed with the GIMIC program^[47]^ using a locally modified version to produce files in cube format.^[74]^ ACID plots for PCT used the ACID program.^[45]^

The underlying research data (molecular geometries, Gaussian input/output files, and input scripts for VMD) are provided via a separate repository.^[75]^

## Results and Discussion

### Benzene

**Figure 2:**
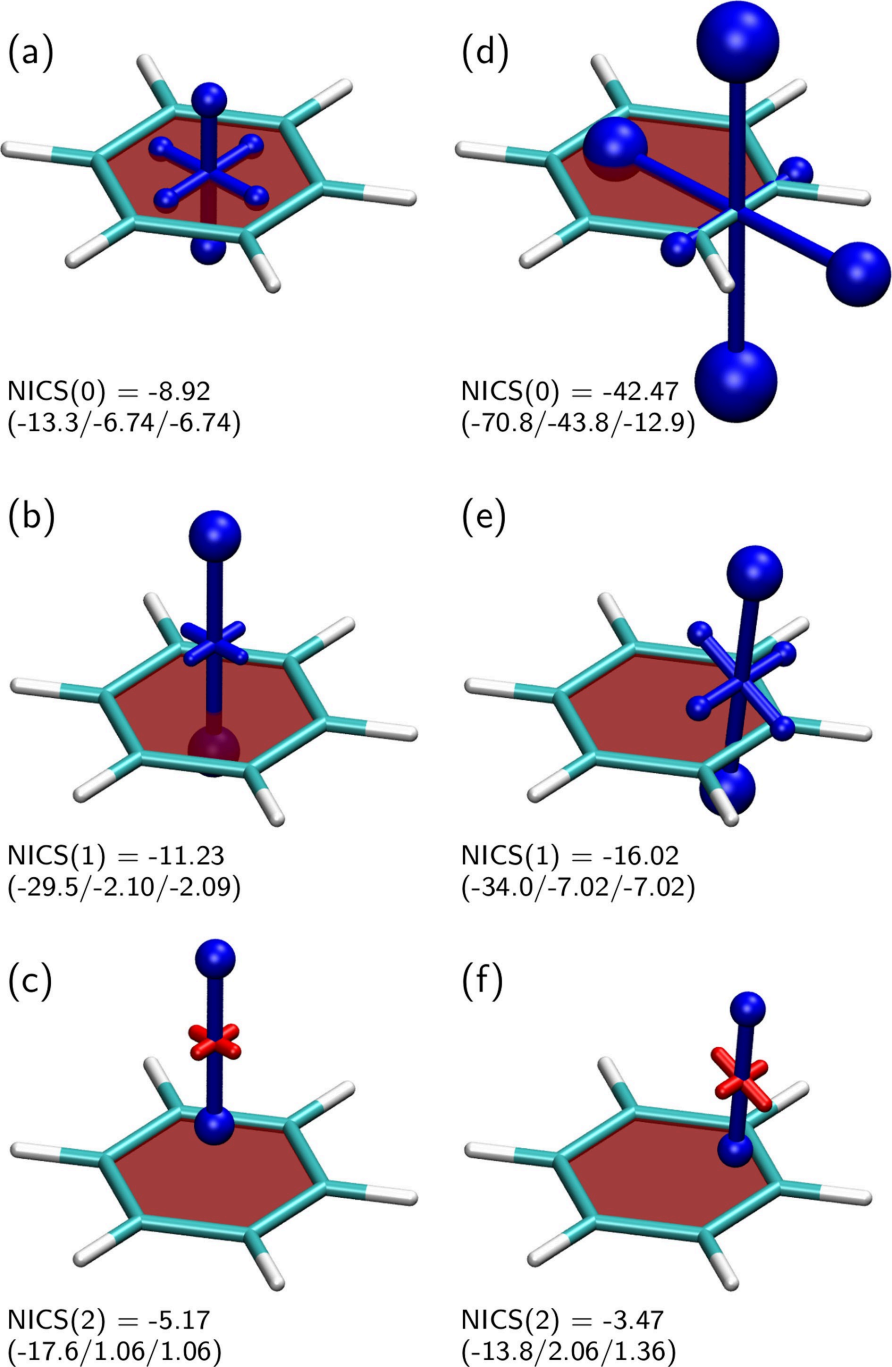
Visualisation of the chemical shielding tensors (VIST) in benzene. Negative (shielded/aromatic) contributions are shown in blue, positive (deshielded/antiaromatic) in red. Below the pictures, isotropic NICS values are given in ppm, the negatives of the associated eigenvalues *t* ^(*i*)^ are shown in parentheses. Shielding tensors were computed at (a) the centre of the molecule, (b) 1 Å and (c) 2 Å above the plane; (d) at the centre of a bond, (e) 1 Å and (f) 2 Å above the bond.

Shielding tensors for benzene were computed at various positions around the molecule to examine how the shielding varies with the position, illustrating the basics of the VIST method (Figure 2). The chemical shielding at the centre of the ring, also denoted NICS(0), is presented in Figure 2a. The tensor representation shows a dominant out‐of‐plane component (‐13.3 ppm) along with two smaller in‐plane components (both −6.7 ppm), which average to an overall isotropic NICS(0) value of −8.9 ppm. Due to the high symmetry present, the out‐of‐plane eigenvalue is equivalent to the NICS(0)_zz_ value, which has been reported as −13.2 ppm elsewhere,^[76]^ in agreement with the present results. Figure 2 (a) clearly shows that appreciable shielding is present along all three coordinate axes, which, in line with Eq. (3), can be understood in the sense that the centre of the ring is surrounded by electron density on all sides (see also Figure S2). To eliminate the influence of the bulk electron density and focus on in‐plane currents in the *π*‐system, the NICS value is often computed 1 or 2 Å above the molecular plane, denoted NICS(1) and NICS(2). The associated tensors are shown in panels (b) and (c), respectively, highlighting that the out‐of‐plane shielding deriving from in‐plane ring currents increases in size whereas the in‐plane shielding almost vanishes (see also Ref. [77]). The dominant contribution to NICS(1) is the out‐of‐plane component of −29.5 ppm, which has been ascribed to the *π*‐electrons^[76]^ and, thus, provides an expedient measure for aromaticity. The NICS(2) tensor is, again, dominated by the out‐of‐plane component and, interestingly, the in‐plane components are slightly deshielded (red). Comparison to the electron density isosurface (Figure S2) shows that the NICS(1) and NICS(2) values are computed well above the main part of the density and it is, thus, understandable that the chemical shielding tensor only ”sees” the ring currents in the aromatic ring rather than the bulk of the *σ*‐electrons.

For comparison, also the shielding tensors along and above one of the CC bonds were computed (Figure 2d–f). It is noteworthy that all NICS(0) eigenvalues are strongly enhanced when computed within the bond. The enhanced contributions can be understood in the sense that a higher electron density yields enhanced diamagnetic shielding according to Eq. (3), but this is not related to aromatic ring currents. An enhancement of NICS values close to the bonds is also visible for NICS(1), in agreement with recent results of 2D and 3D scans.[^25^, ^57^] This enhancement is lost in the case of the NICS(2) tensor in Figure 2.

In summary, the above discussion highlights the importance of local variations in the overall chemical shielding and its individual tensor components. The VIST method proved to be an expedient method to visualise both phenomena.

### Phenanthrene – variations in local aromaticity

**Figure 3:**
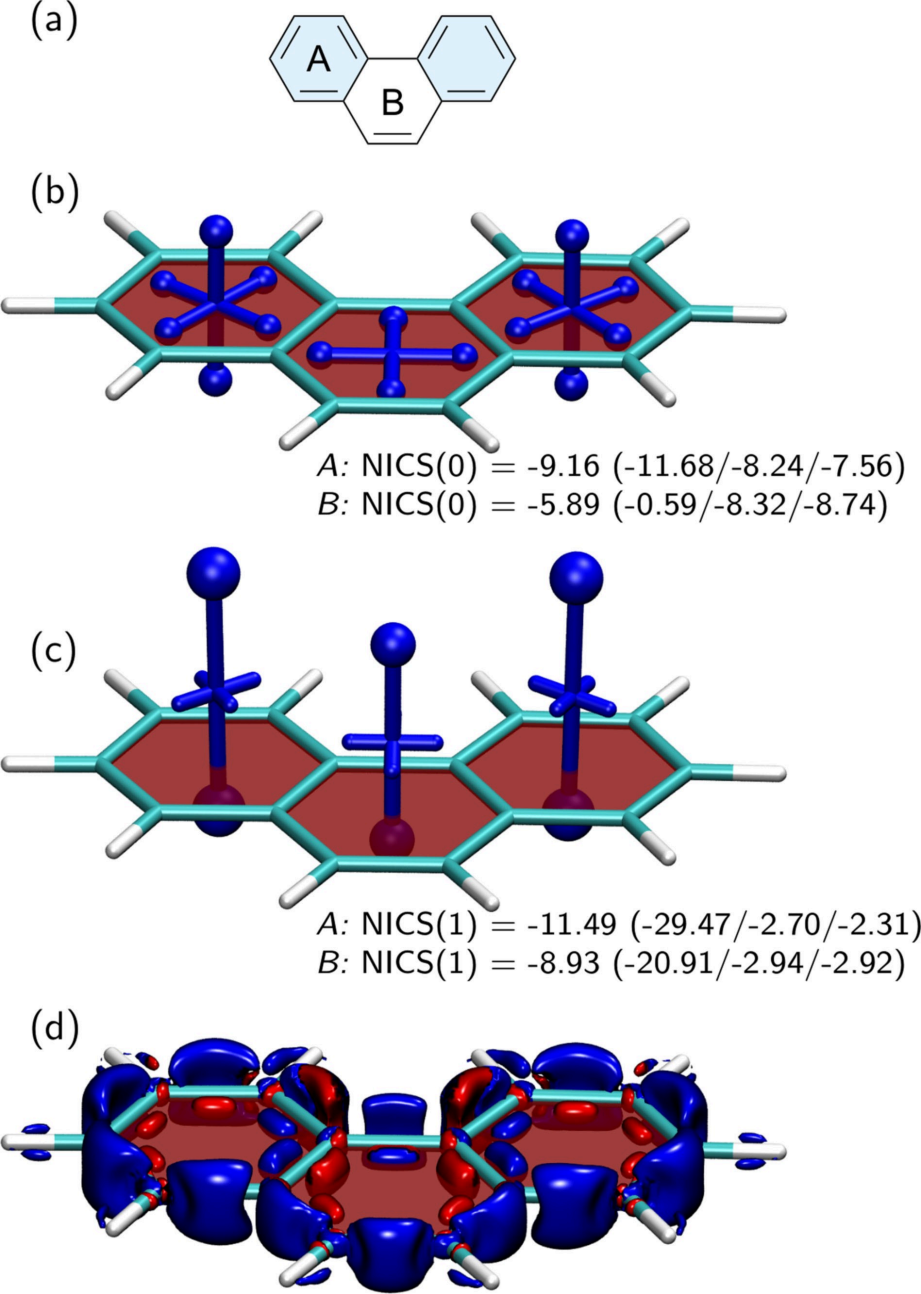
Analysis of local aromaticity in phenanthrene: (a) molecular structure with Clar sextets highlighted in blue; visualisation of the chemical shielding tensors (VIST, see Figure 2 for details) computed at (b) the centre of each ring and (c) 1 Å above the plane. NICS values (in ppm) are reported for the outer (*A*) and inner (*B*) rings. The isosurface of the current density modulus induced by a magnetic field in z‐direction is shown in (d) (cutoff 0.1 a.u., blue ‐ diatropic, red ‐ paratropic).

Next, we consider the phenanthrene molecule as an example of a polycyclic aromatic hydrocarbon with the goal of studying variations in its local aromaticity. The molecular structure of phenanthrene is shown in Figure 3a. Here, we highlight the two Clar sextets on the outer rings, noting that this is the only possibility of creating a resonance structure with two disjoint sextets and one, thus, expects the outer rings to have enhanced local aromaticity.^[78]^ Indeed, when considering the NICS(0) values in Figure 3b, we find that the outer rings (*A*) experience significantly enhanced shielding when compared to the inner ring (*B*). Interestingly, the out‐of‐plane component of NICS(0) computed at the inner ring almost vanishes. A comparison to benzene shows that the isotropic NICS(0) value at the outer rings is slightly higher for phenanthrene than benzene (−9.2 *vs* −8.9 ppm) but that the out‐of‐plane component is clearly lower (−11.7 ppm *vs* −13.3 ppm).

To reduce any contributions from the *σ*‐system, we proceed to the NICS(1) tensors, as shown in Figure 3c. These have strong out‐of‐plane components in all three rings, representing strong in‐plane aromaticity and, again, the outer rings are notably enhanced. The isotropic NICS(1) values, as well as the individual tensor components of the outer rings, are very similar to benzene (Figure 2b) with the exception that the eigenvectors corresponding to the smaller contributions are slightly tilted out of plane.

For comparison, we also want to show a different representation of aromaticity,^[46]^ which proceeds by computing the current density induced by a magnetic field perpendicular to the molecular plane and dividing the current into diatropic and paratropic contributions, which can roughly be understood as the currents giving rise to diamagnetic shielding [Eq. (3)] and paratropic deshielding [Eq. (4)], respectively. Diatropic and paratropic ring currents are shown in Figure 3d in blue and red. In line with previous results,[^46^, ^47^] one finds diatropic ring currents outside of the bonds, extending over *σ*‐ and *π*‐orbitals, and paratropic currents inside, deriving mainly from *σ*‐orbitals. The diatropic currents (blue) dominate, explaining the net shielding seen in Figure 3b and Figure 3c. Closer inspection shows that the main paratropic contributions are located within the inner ring, explaining why shielding is reduced there, in particular for the NICS(0) tensors. The effect of these *σ*‐contributions is reduced once one moves out of the molecular plane, explaining why strong shielding is obtained for all NICS(1) tensors.

In summary, we found that for a simple system like phenanthrene the isotropic NICS values already reflect the correct trends in terms of its variations in local aromaticity. However, the VIST method provides a detailed and intuitive representation of the individual shielding components, including the somewhat surprising result that the out‐of‐plane component at the inner ring almost vanishes. We have also highlighted that the visualisation of the current density can provide complementary information to the shielding tensors, thus, providing a combined strategy for illuminating intricate details of aromatic ring currents.

### Cyclobuta[l]phenanthrene – antiaromaticity and Baird aromaticity

Whereas the previous two molecules were aromatic, we want to proceed by illustrating how the VIST method can be used particularly effectively to study the interplay of local aromaticity and antiaromaticity and their modulation via the electronic spin state. For this purpose, we add a cyclobutadiene (CBD) ring to phenanthrene to produce the cyclobuta[l]phenanthrene molecule. This molecule was initially synthesised by Anhalt et al.^[79]^ More recently, it was reported as a potential singlet fission chromophore, owing to its low‐energy first triplet state (*<*1 eV) despite maintaining a large excitation energy of its first singlet excited state (*>*2 eV).^[6]^ The low triplet energy was explained by the combination of ground‐state antiaromaticity and triplet state Baird aromaticity of the CBD ring and we shall illustrate these phenomena here.

**Figure 4:**
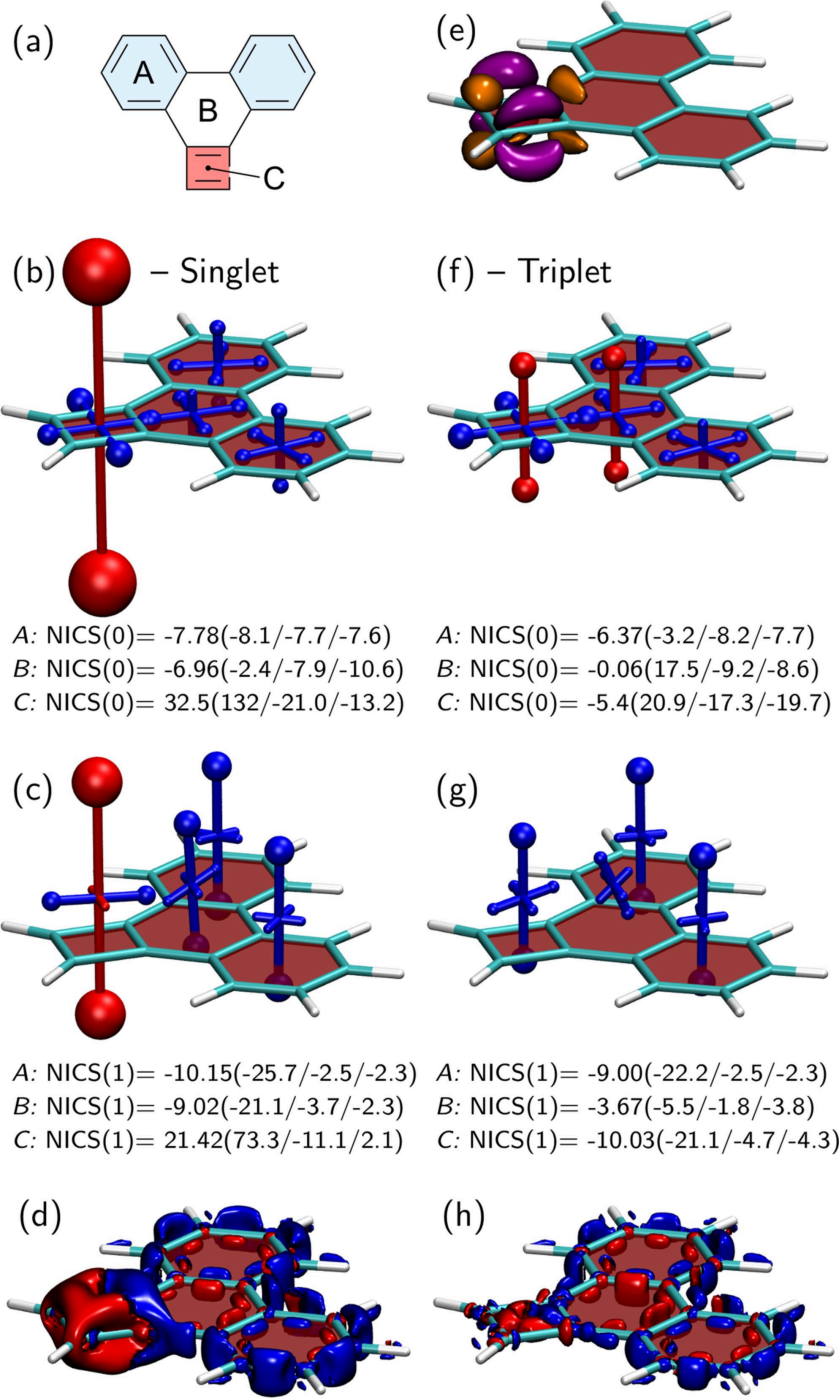
Analysis of local (anti)aromaticity in the lowest singlet and triplet states of cyclobuta[l]phenanthrene: (a) molecular structure with Clar sextets highlighted in blue and the antiaromatic ring in red; visualisation of the chemical shielding tensors (VIST, see Figure 2 for details) computed at (b/f) the centre of each ring and (c/g) 1 Å above the plane for the singlet/triplet; (e) difference density between singlet and triplet (cutoff 0.005 a.u.); current density modulus induced by a magnetic field in z‐direction (d/h) for the singlet/triplet (cutoff 0.1 a.u.). NICS values (in ppm) are reported for the *A*, *B*, *C* positions as shown in (a).

The molecular structure of cyclobuta[l]phenanthrene is shown in Figure 4a, highlighting its aromatic Clar sextets in blue along with its antiaromatic CBD ring in red. The NICS(0) tensors of the singlet ground state presented in Figure 4b have a striking appearance with a strongly dominant out‐of‐plane antiaromatic (red) component on the CBD ring. The associated eigenvalue of 132 ppm is far higher in magnitude than any shielding value found in the above examples. Moving to the NICS(1) tensors in Figure 4c we find that antiaromaticity is still clearly visible but that the magnitude of the deshielding is strongly reduced (from 132 to 73 ppm for the dominant eigenvalue), a result also obtained for an isolated CBD molecule.[^77^, ^80^] When viewing the phenanthrene part of the molecule, we find that the shielding tensors are only slightly perturbed as compared to isolated phenanthrene (Figure 3).

Following Baird's rule,^[31]^ one expects the four‐membered CBD ring to exhibit aromaticity in its lowest triplet state. To examine this hypothesis, we have performed computations on this state. We start the discussion with the difference in electron density between singlet and triplet to describe the electronic rearrangement involved. Figure 4e shows that the difference density is located on the CBD ring and, specifically, that the transition from the singlet to the triplet means a reduction (orange) in density along the bonds that are indicated as double bonds in Figure 4a and an enhancement (purple) in density on the other two bonds, resulting in an overall more even charge distribution around the CBD ring.

The change from singlet to triplet has a profound impact on the magnetic shielding, as shown in Figure 4f and g, most importantly by eliminating the strongly deshielded component perpendicular to the CBD ring (*C*). Viewing the NICS(0) tensors (Figure 4f), we find that the CBD ring remains slightly deshielded while also the central 6‐membered ring (*B*) obtains a slightly deshielded contribution. The z‐components of the shielding tensors on the outer rings (*A*) are also reduced in magnitude, yielding a NICS_zz_(0) value of only −3.2 ppm. Interpreting the NICS(0) contributions is not straightforward as it is not clear how to separate between the contributions from the *π*‐electrons in the individual rings as well as the *σ*‐electrons. Therefore, we also present the NICS(1) contributions to get a different viewpoint (Figure 4g). Interestingly, the NICS(1) tensors all show pronounced aromaticity for the CBD ring as well as the outer phenanthrene rings. It is noteworthy here that the values for the outer rings in phenanthrene (*A*) are almost unaltered between the singlet and triplet states, i. e. when comparing panels (c) and (g), suggesting that their *π*‐electrons are not strongly perturbed by the excitation. The central ring (*B*), on the other hand, experiences a significant decrease in aromaticity. In summary, Figure 4g suggests viewing the electronic structure of cyclobuta[l]phenanthrene in its triplet state as a combination of a Baird aromatic quartet with two Clar sextets (cf. Ref. 81).

Before concluding, we also want to present current density isosurfaces^[46]^ for the singlet and triplet states. In the singlet state (Figure 4d) one finds a similar pattern around the phenanthrene part of the molecule as seen for isolated phenanthrene, cf. Figure 3d. However, pronounced current densities are seen around the CBD part in line with the pronounced shielding tensors. In Figure 4d, the current on the outer part of CBD is shown as paratropic (red) whereas the inner part is shown as diatropic (blue). This does not mean that half of the CBD unit is in fact aromatic, but it just derives from the fact that the assignment as paratropic and diatropic depends on the gauge origin, which lies outside the CBD ring in the present case. Moving to the triplet, Figure 4h, one finds that the pronounced currents at the CBD ring disappear and that currents around the phenanthrene part are slightly modulated.

Finally, we note that strongly antiaromatic systems with emerging biradical character and low triplet energies would require a multireference treatment[^82^, ^83^] for a reliable description of the wavefunctions involved, noting that NICS values and current densities may indeed be strongly affected by multireference effects.[^80^, ^84^, ^85^] Nonetheless, we believe that Figure 4 provides a good, semiquantitative description of the relevant physics.

### Paracyclophanetetraene – interplay of local aromaticity and global antiaromaticity in a macrocycle

**Figure 5:**
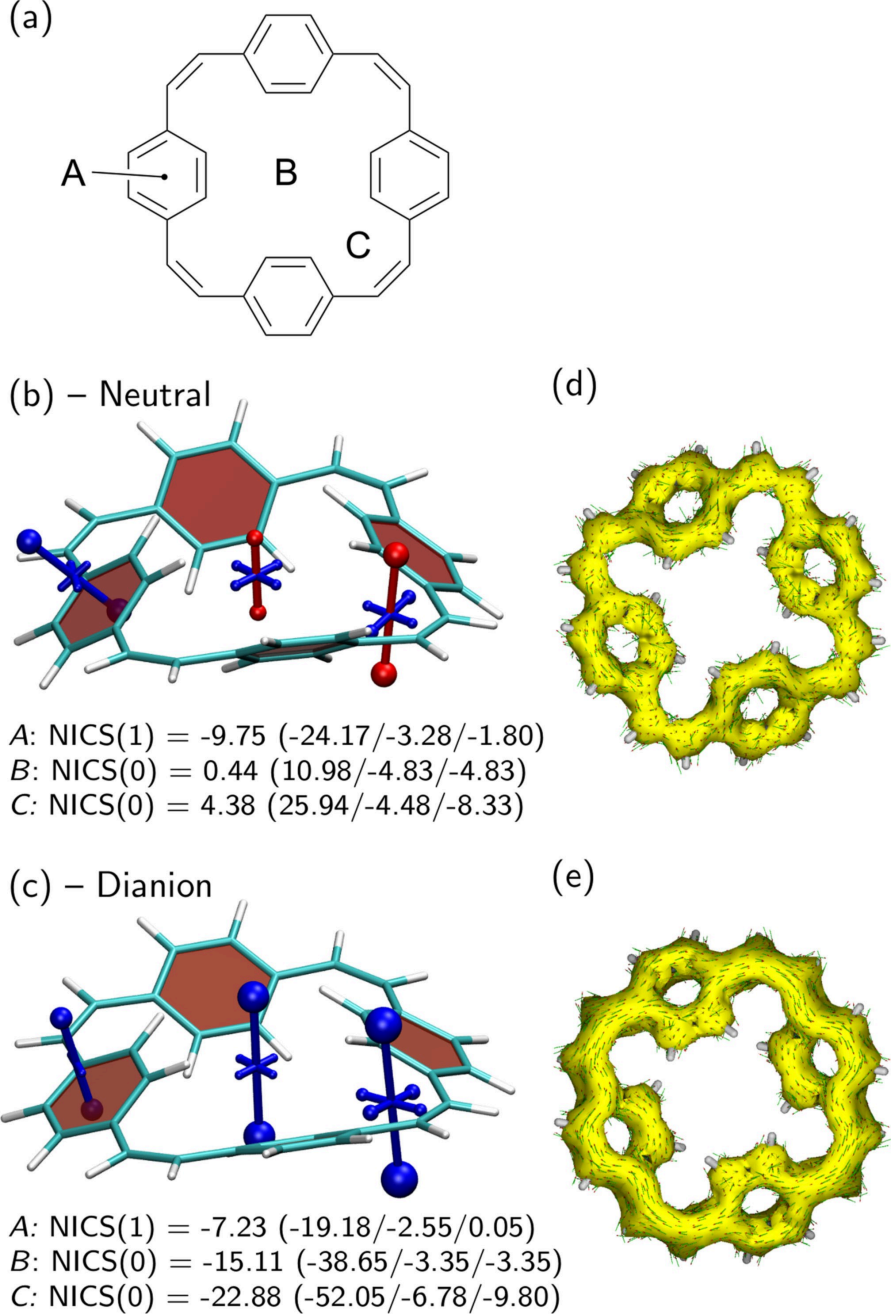
Analysis of local and global (anti)aromaticity in paracyclophanetetraene: (a) molecular structure; visualisation of the chemical shielding tensors (VIST, see Figure 2 for details) for (b) the neutral and (c) the dianion – NICS values (in ppm) are reported for the phenylene ring (*A*), the centre (*B*), and side (*C*) of the macrocycle; ACID plots for (d) the neutral and (e) the doubly reduced form (isovalue 0.05).

Paracyclophanetetraene (PCT)[^23^, ^24^] is a non‐planar macrocycle with competing *π*‐conjugated systems aligned in different planes and, hence, a particularly challenging case to visualise and quantify its local variations in (anti)aromaticity. The macrocycle, as shown in Figure 5a, features a *π*‐conjugated perimeter of 24 [4*n*] *π*‐electrons as well as four slightly twisted phenylene subunits with 6 [4*n*+2] *π*‐electrons, each. In the neutral state, the macrocycle is formally antiaromatic but, in practice, was reported to not exhibit any measurable antiaromatic properties.^[24]^ Twofold reduction, on the other hand, was reported to create a globally aromatic macrocyclic system of [4*n*+2] *π*‐electrons[^23^, ^24^] whose enhanced stability renders PCT a promising new material for sodium‐ion battery anodes.

Chemical shielding tensors were computed at three positions within the macrocycle: 1 Å off the plane of one of the phenylene rings (*A*), at the centre of the macrocycle (*B*), and on the side next to the double bond (*C*). Starting with the centre position (*B*), we find a deshielded component of 11.0 ppm along with two shielded components (‐4.8 ppm) averaging to an isotropic NICS value close to zero. Moving closer to the side of the macrocycle (*C*) we find somewhat stronger deshielding (25.9 ppm), supporting the presence of global antiaromaticity for the macrocycle. However, it should be pointed out that part of the deshielding derives from diatropic currents in the benzene rings as shown in Figure S3, thus, indicating only weak antiaromatic character in line with the experimental observations.

Next, we test the hypothesis of local aromaticity on the phenylene rings. For this reason, we have computed the NICS(1) tensor for one of the phenylene rings shown on the left in Figure 5b. The tensor is tilted to be perpendicular to the phenylene ring but otherwise possesses a similar shape to the NICS(1) tensor of an isolated benzene molecule (Figure 2b) with only slightly reduced aromaticity (−24.2 *vs* −29.5 ppm for the dominant eigenvalue). This highlights that the local aromaticity in the phenylene rings is largely unperturbed by the macrocycle. Note that the tilt seen in Figure 5b represents a non‐trivial combination of all the tensor components and would be difficult to comprehend without the VIST method.

An analysis of the dianion is presented in Figure 5c. Viewing first the geometry, one finds that the overall system slightly planarises. In addition, the formal vinylene double bond elongates and becomes more distorted out‐of‐plane as measured by the dihedral angle involving the adjacent carbon atoms (see Table S1). By contrast, the single bond connecting the phenylene and vinylene units shortens and planarises. Both phenomena indicate that the molecule optimises for enhanced conjugation across the perimeter enabling stronger global ring currents (see Ref. [19] for a similar discussion on cycloparaphenylenes). The chemical shielding tensors of the dianion, shown in Figure 5c, have a dramatically different appearance when compared to the neutral state. Strong aromaticity (blue) perpendicular to the plane of the macrocycle is found for all three positions probed. The dominant eigenvalues obtained for positions *B* and *C*, −38.7 and −52.1 ppm, are even higher than the NICS(1)_zz_ eigenvalue for benzene, shown in Figure 2b. Viewing the phenylene position (*A*) one finds somewhat reduced shielding values and a tilt of the main component with respect to Figure 5b, making it almost perpendicular to the plane of the macrocycle. These findings indicate that the local aromaticity of the phenylene ring is perturbed to allow for enhanced global aromaticity.

For comparison, we also want to show the ACID plots^[45]^ of the neutral and doubly reduced form, Figure 5d and e. The isosurfaces shown represent the delocalised electrons whereas the small arrows represent the direction of the current if a magnetic field is applied in z‐direction. The ACID plots show that electron delocalisation extends over all carbon atoms in the macrocycle in both states shown and that there is more delocalisation in the dianion than in the neutral state. Upon closer inspection, one finds arrows going in a clockwise direction in Figure 5e, representing the diatropic ring current that is responsible for the strong shielding seen in Figure 5c. It is not possible to identify the paratropic ring currents in panel (d) that are responsible for the deshielding found in the neutral state. It is even challenging to locate the local diatropic ring currents in the phenylene units that give rise to their local aromaticity, which is unambiguously seen in Figure 5b.

In summary, Figure 5 underscores the dramatic changes PCT undergoes upon twofold reduction, explaining its remarkable redox properties. The ACID plots were useful for highlighting the overall electron distribution but the new VIST method provided a direct representation of the remarkable changes in electronic structure following the switch from local to global aromaticity.

### Cycloparaphenylene

Cycloparaphenylenes (CPP), composed of phenylene units connected in *para*‐position, are an intensively investigated class of conjugated macrocycles.[^19^, ^20^, ^86^, ^87^] CPPs are attracting interest due to their unique optoelectronic properties[^88^, ^89^, ^90^] in combination with their rich host‐guest chemistry^[91]^ providing a promising basis for applications from solid‐state nanomaterials to biological imaging.^[92]^ When viewing the global properties of CPP macrocycles, every phenylene unit contributes 4 *π*‐electrons to the macrocyclic *π*‐conjugated pathway, meaning that any [*n*]CPP (where *n* represents the number of phenylene units) has 4*n* electrons in its *π*‐conjugated pathway and is, thus, expected to be antiaromatic. Two‐fold oxidation or reduction creates global aromaticity,[^19^, ^20^] in line with the discussion on PCT in the last section.

**Figure 6:**
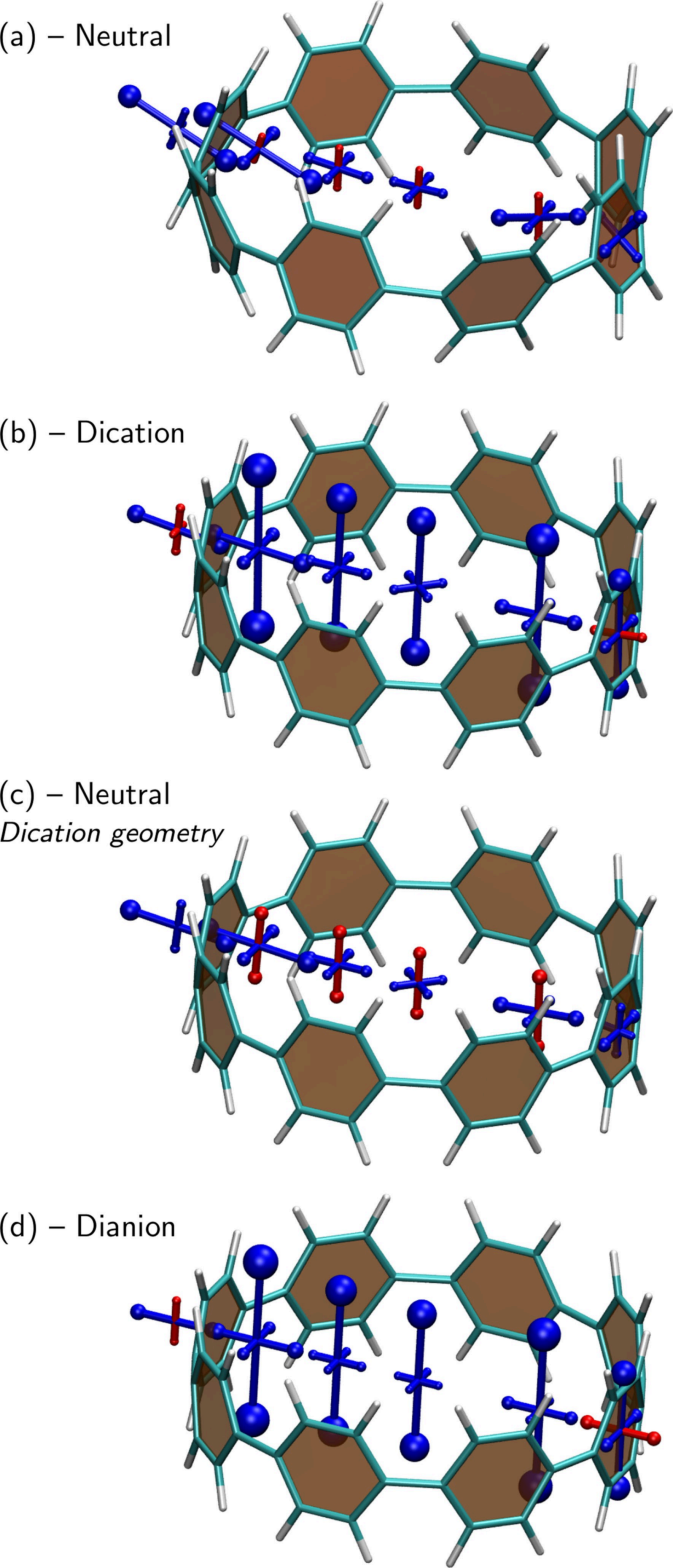
Analysis of local and global (anti)aromaticity in [8]CPP. Visualisation of the chemical shielding tensors (VIST, see Figure 2 for details) for (a) the neutral form, (b) the dication, (c) the neutral form at the dication geometry, (d) the dianion.

An analysis of the neutral molecule is presented in Figure 6a. Chemical shielding tensors were computed along a line going from the centre of one phenylene ring to the centre of a phenylene ring on the opposite side, essentially performing a NICS‐XY scan^[55]^ through the macrocycle. Inside the macrocycle, one finds a slight deshielding (red) of up to 3.7 ppm in the out‐of‐plane direction. However, this is compensated by in‐plane shielding and the isotropic NICS values inside the ring are all negative, in agreement with Ref. 20. When chemical shielding tensors are computed close to a phenylene ring, as shown on the left, one finds that they are strongly tilted as these now represent local aromaticity in the phenylene rings, which lie perpendicular to the plane of the macrocycle.

Next, we present computations on the dication, which was reported to exhibit strong global aromaticity.^[20]^ Indeed, viewing Figure 6b, we find strongly shielded (blue) tensor components perpendicular to the plane of the macrocycle. At the centre of the ring, we find an out‐of‐plane NICS component of −36.4 ppm, which is similar to the dianion of PCT as shown in Figure 5c, and slightly higher values (up to −51.6 ppm) are obtained closer to the phenylene rings. Viewing the contributions to the left and right of the phenylene ring shown on the left in Figure 6b, one finds that the local aromaticity on the phenylene ring is clearly reduced as opposed to the neutral form, suggesting that the redox chemistry of [8]CPP can be described in terms of a switch between local and global aromaticity similarly to PCT.

Figure 6a shows that there is no appreciable global antiaromaticity for [8]CPP at its ground state optimised geometry. However, it is interesting to probe whether [8]CPP has a propensity toward antiaromaticity in principle. For this purpose, we have performed computations of the neutral system at the dication geometry. The dication has shorter CC bonds (1.45 *vs* 1.48 Å) and reduced torsion angles (7° *vs* 30°) between adjacent phenylene rings, allowing for enhanced conjugation throughout the macrocycle. The resulting shielding tensors, shown in Figure 6c, highlight that, indeed, the dication geometry leads to enhanced antiaromaticity in the neutral system with NICS components up to 10.3 ppm. We are, thus, left to conclude that the neutral molecule possesses a propensity towards antiaromaticity due to its macrocyclic [4*n*] *π*‐electron system but that it has sufficient conformational flexibility to adopt a geometry where antiaromaticity is minimised.

Finally, viewing the dianion in Figure 6d, we find that its chemical shielding structure looks very similar to Figure 6b, highlighting that it exhibits global aromaticity just as the dication.

### Norcorrole dimer – through‐space aromaticity

**Figure 7:**
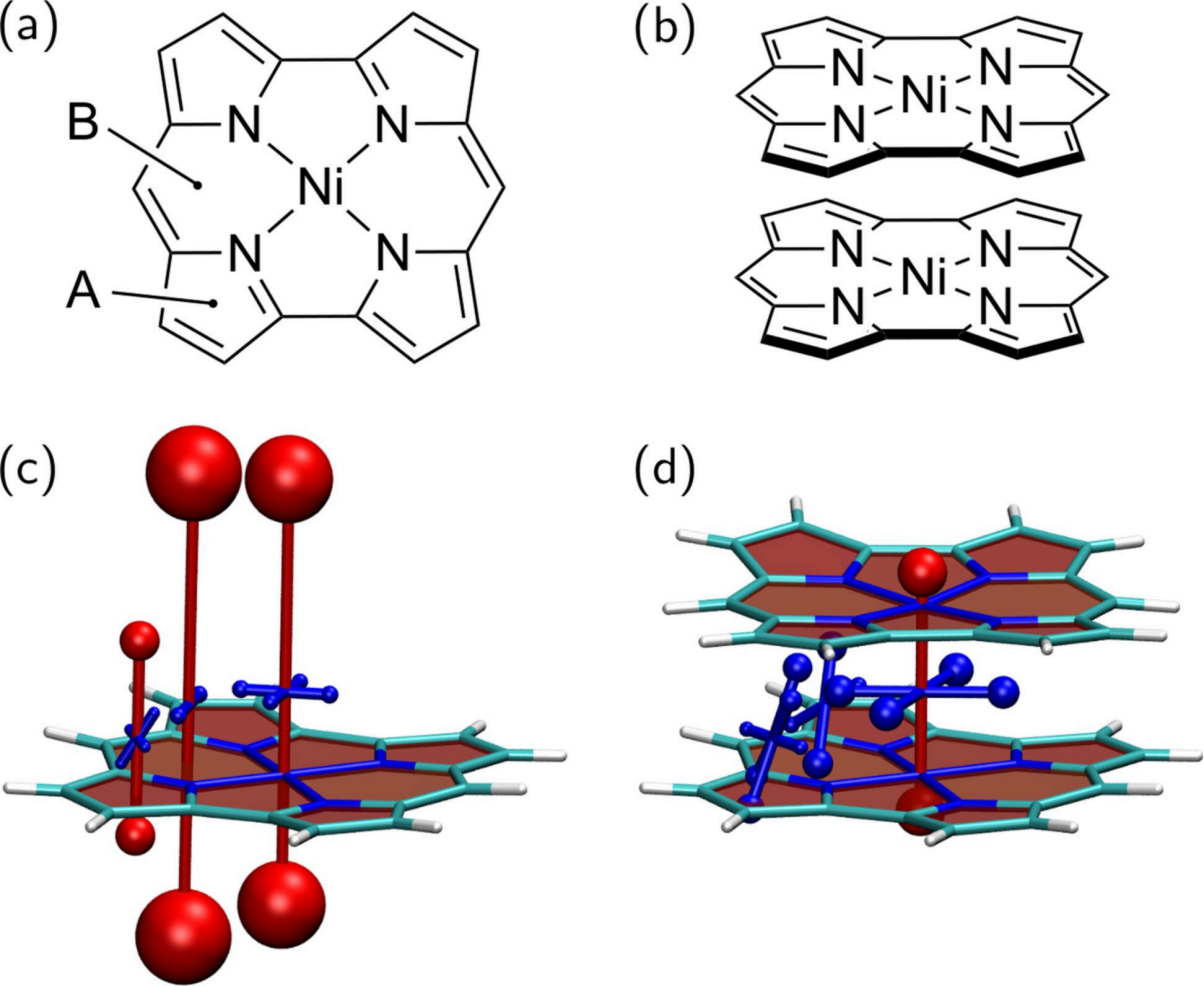
Analysis of local (anti)aromaticity in a stacked norcorrole dimer: Molecular structures of (a) the monomer and (b) the dimer; visualisation of the chemical shielding tensors (VIST, see Figure 2 for details) for (c) the monomer and (d) the dimer.

Ni(II) norcorrole (Figure 7a) is a prominent antiaromatic compound. Its [4*n*] *π*‐electron system cannot escape planarity due to the rigidity of the molecular structure, explaining why strong antiaromaticity is indeed observed in this molecule. Bulky mesityl substituents can be attached to the conjugated core of Ni(II) norcorrole to improve its stability,^[93]^ but the compound is comparatively stable also without these substituents, despite the antiaromaticity.^[94]^ The comparatively high stability of Ni(II) norcorrole makes it an ideal molecule to study stacking interactions between antiaromatic *π*‐conjugated systems and the ensuing emergence of three‐dimensional aromaticity. Initially, stacking of flexibly linked Ni(II) norcorrole complexes was investigated,^[37]^ followed by a more recent report of a rigid cyclophane composed of two Ni(II) norcorrole units and two bithiophene linkers.^[38]^ These studies highlighted the importance of through‐space currents connecting the two macrocycles.

Within this work, we study the Ni(II) norcorrole complex reported in Ref. 38 but with removed bithiophene linkers, as shown in Figure 7b. Shielding tensors were computed at three different positions, as shown in Figure 7a: 1 Å above the outer 5‐membered ring (*A*), 1 Å above the 6‐membered ring (*B*), and at the centre of the complex, halfway between the two Ni‐atoms (denoted Ni). Starting with the Ni(II) norcorrole monomer (Figure 7c) we find pronounced antiaromaticity when measured at the Ni and *B* positions with dominant eigenvalues of 185 and 155 ppm, respectively, which are higher than any of the values seen in the above examples. Interestingly, the 5‐membered ring (*A*) shows significantly reduced antiaromaticity. This reduction in deshielding for the 5‐membered ring, which was also seen in Ref. 26, has been explained in the sense that antiaromatic currents are only present on the inner part of the ring and that norcorrole possesses an aromatic pathway on the perimeter.^[25]^

To study the effect of stacking between two norcorrole units, we have added a second ring to the system but, otherwise, left the geometry of the first ring and the positions of the chemical shielding tensors unchanged. The striking impact of the second ring is illustrated in Figure 7d. The antiaromaticity disappears for positions *A* and *B* and is strongly reduced at the centre of the complex.

Viewing only the isotropic NICS value at the centre of the ring (1.2 ppm) it might be tempting to jump to the conclusion that no relevant shielding effects are present. However, the VIST plot immediately shows that this is incorrect and that the isotropic value is obtained as an average of strong out‐of‐plane deshielding (49 ppm) and strong in‐plane shielding (−24 and −21 ppm). Indeed, both in‐plane tensor components show a four‐fold increase when compared to the isolated monomer. Their values lie between the NICS(0)_zz_ and NICS(1)_zz_ values for benzene (Figure 2), thus, indicating the presence of strong aromatic currents. It is not possible that these currents run within the molecular planes, since the observed shielding lies in parallel to the molecular planes. Therefore, the strong in‐plane components are a direct indication of through‐space currents flowing between the two norcorroles, as shown explicitly in Ref. 38.

Closer inspection of the tensors at locations *A* and *B* (see also Figure S4) shows that these have an unusual shape with their main components tilted with respect to the molecular planes and strong non‐orthogonality among the three principal axes of the chemical shielding tensor. We do not attempt to identify the individual ring currents responsible for these results but want to point out that the non‐standard electronic structure properties of the norcorrole dimer are reflected by these tensors.

### Scope and Limitations

We have shown that the VIST method provides a compact and intuitive representation of the optoelectronic properties of a diverse set of polycyclic systems. The method proved to be particularly suitable for probing electron delocalisation occurring in different molecular planes where existing methods are hard to apply. On the other hand, we want to point out that – just like any other computational method – shielding tensors can be misused or overinterpreted. Certainly, the practice of computing a limited number of NICS tensors at the centres of the rings, as done above, does not provide a complete representation of the underlying ring‐currents.[^95^, ^96^] More fine‐grained NICS scans or a more detailed study of currents at individual locations may be required to elucidate all of the subtleties. However, if the aim is to provide an intuitive representation of the most pronounced changes in electronic structure following changes in the charge or spin state, then VIST provides a useful and powerful yet lightweight computational tool.

## Conclusions

We can conclude that the visualisation of chemical shielding tensors (VIST) presented in this work is a highly valuable method to elucidate aromaticity and antiaromaticity in molecules. The VIST method shows the chemical shielding tensor at various points in space around a molecule, thus, allowing to represent the local variations and anisotropy of the chemical shielding in unprecedented clarity. VIST is, thus, particularly useful for large, non‐planar molecular systems where it can be exceedingly difficult to obtain the required information otherwise. VIST also provides an expedient route to illustrate the influence of different electronic charge and spin states on aromaticity and antiaromaticity.

Within this work, the basic properties of the VIST method could be illustrated in the case of planar polycyclic hydrocarbons. Starting with benzene, we investigated how the shape of the shielding tensor changes at different positions surrounding the molecule. Moving to phenanthrene, we illustrated local variations in aromaticity in this molecule and discussed the shielding tensors in the context of the magnetically induced currents. For cyclobuta[l]phenanthrene the interplay of local aromaticity and antiaromaticity in its singlet state and the emergence of Baird aromaticity in its triplet was studied.

Moving to non‐planar macrocycles with competing aromatic and antiaromatic *π*‐systems aligned in different planes, we were able to illuminate the full power of the VIST method. Starting with PCT, we highlighted the combination of local aromaticity and weak global antiaromaticity in its ground state and a switch to global aromaticity in its doubly reduced state. A similar picture was obtained for [8]CPP, which exhibits pronounced aromatic character in its doubly oxidised and reduced states. The VIST method, thus, provided a clear explanation of the remarkable redox properties of both macrocycles. Finally, studying the stacking between two norcorrole molecules, we showed how strong in‐plane antiaromaticity in Ni(II) norcorrole was replaced by weaker in‐plane antiaromaticity in combination with through‐space aromaticity upon stacking.

Practically, VIST builds on the computation of NICS values and can, thus, be carried out whenever NICS values are available. Moreover, it is readily integrated into existing tools used for the visualisation of molecular structures and electron densities. Therefore, we are convinced that VIST constitutes a powerful but also flexible and lightweight computational tool and provides a much‐needed extension to the toolbox of chemists studying (anti)aromaticity or using it in their molecular design.

## Conflict of interest

The authors declare no conflict of interest.

## Supporting information

As a service to our authors and readers, this journal provides supporting information supplied by the authors. Such materials are peer reviewed and may be re‐organized for online delivery, but are not copy‐edited or typeset. Technical support issues arising from supporting information (other than missing files) should be addressed to the authors.

Supplementary

## References

[1] M. Faraday , Philos. Trans. R. Soc. London 1825, 115, 440–466.

[2] M. B. Smith , J. Michl , Chem. Rev. 2010, 110, 6891–936. 21053979 10.1021/cr1002613

[3] J. Stoycheva , A. Tadjer , M. Garavelli , M. Spassova , A. Nenov , J. Romanova , J. Phys. Chem. Lett. 2020, 11, 1390–1396. 31971385 10.1021/acs.jpclett.9b03406

[4] M. Pinheiro , F. B. C. Machado , F. Plasser , A. J. A. Aquino , H. Lischka , J. Mater. Chem. C 2020, 8, 7793–7804.

[5] K. J. Fallon , P. Budden , E. Salvadori , A. M. Ganose , C. N. Savory , L. Eyre , S. Dowland , Q. Ai , S. Goodlett , C. Risko , D. O. Scanlon , C. W. M. Kay , A. Rao , R. H. Friend , A. J. Musser , H. Bronstein , J. Am. Chem. Soc. 2019, 141, 13867–13876. 31381323 10.1021/jacs.9b06346

[6] O. El Bakouri , J. R. Smith , H. Ottosson , J. Am. Chem. Soc. 2020, 142, 5602–5617. 32107921 10.1021/jacs.9b12435 PMC7307911

[7] L. Wang , L. Lin , J. Yang , Y. Wu , H. Wang , J. Zhu , J. Yao , H. Fu , J. Am. Chem. Soc. 2020, 142, 10235–10239. 32437140 10.1021/jacs.0c00089

[8] E. Clar, The aromatic sextet, Wiley, London, **1972**.

[9] Z. Sun , Z. Zeng , J. Wu , Acc. Chem. Res. 2014, 47, 2582–2591. 25068503 10.1021/ar5001692

[10] A. Das , T. Müller , F. Plasser , H. Lischka , J. Phys. Chem. A 2016, 120, 1625–1636. 26859789 10.1021/acs.jpca.5b12393 PMC4789636

[11] F. Hinkel , J. Freudenberg , U. H. F. Bunz , Angew. Chem. Int. Ed. 2016, 55, 9830–9832; 10.1002/anie.201605490 27348797

[12] S. N. Intorp , M. Hodecker , M. Müller , O. Tverskoy , M. Rosenkranz , E. Dmitrieva , A. A. Popov , F. Rominger , J. Freudenberg , A. Dreuw , U. H. Bunz , Angew. Chem. Int. Ed. 2020, 59, 12396–12401; 10.1002/anie.201915977 PMC7384067 32190951

[13] I. Benkyi , O. Staszewska-Krajewska , D. T. Gryko , M. Jaszunski , A. Stanger , D. Sundholm , J. Phys. Chem. A 2020, 124, 695–703. 31917567 10.1021/acs.jpca.9b11315

[14] T. Dumslaff , Y. Gu , G. M. Paterno , Z. Qiu , A. Maghsoumi , M. Tommasini , X. Feng , F. Scotognella , A. Narita , K. Müllen , Chem. Sci. 2020, 1, 12816–12821. 10.1039/d0sc04649c PMC8163021 34094476

[15] M. D. Peeks , M. Jirasek , T. D. W. Claridge , H. L. Anderson , Angew. Chem. Int. Ed. 2019, 58, 15717–15720; 10.1002/anie.201909032 PMC6856819 31397538

[16] M. Rickhaus , M. Jirasek , L. Tejerina , H. Gotfredsen , M. D. Peeks , R. Haver , H.-W. Jiang , T. D. W. Claridge , H. L. Anderson , Nat. Chem. 2020, 12, 236–241. 31959963 10.1038/s41557-019-0398-3 PMC7049293

[17] Y. Han , S. Dong , J. Shao , W. Fan , C. Chi , Angew. Chem. Int. Ed. 2021, 60, 2658–2662. 10.1002/anie.202012651 33047813

[18] G. V. Baryshnikov , R. R. Valiev , R. T. Nasibullin , D. Sundholm , T. Kurten , H. Ågren , J. Phys. Chem. A 2020, 124, 10849–10855. 33301674 10.1021/acs.jpca.0c09692 PMC7770816

[19] S. Taubert , D. Sundholm , F. Pichierri , J. Org. Chem. 2010, 75, 5867–5874. 20701321 10.1021/jo100902w

[20] N. Toriumi , A. Muranaka , E. Kayahara , S. Yamago , M. Uchiyama , J. Am. Chem. Soc. 2015, 137, 82–85. 25526281 10.1021/ja511320f

[21] R. Ayub , O. El Bakouri , J. R. Smith , K. Jorner , H. Ottosson , J. Phys. Chem. A 2021, 125, 570–584. 33427474 10.1021/acs.jpca.0c08926 PMC7884009

[22] Z. Li , T. Y. Gopalakrishna , Y. Han , Y. Gu , L. Yuan , W. Zeng , D. Casanova , J. Wu , J. Am. Chem. Soc. 2019, 141, 16266–16270. 31565929 10.1021/jacs.9b09780

[23] W. Huber , K. Müllen , O. Wennerström , Angew. Chem. Int. Ed. 1980, 19, 624–625;

[24] S. Eder , D. J. Yoo , W. Nogala , M. Pletzer , A. Santana Bonilla , A. J. White , K. E. Jelfs , M. Heeney , J. W. Choi , F. Glöcklhofer , Angew. Chem. Int. Ed. 2020, 59, 12958–12964; 10.1002/anie.202003386 PMC7496320 32368821

[25] P. B. Karadakov , Org. Lett. 2020, 22, 8676–8680. 33104371 10.1021/acs.orglett.0c03254

[26] S. Y. Liu , H. Kawashima , N. Fukui , H. Shinokubo , Chem. Commun. 2020, 56, 6846–6849. 10.1039/d0cc02543g 32432636

[27] R. R. Valiev , L. I. Valiulina , H. Fliegl , D. Sundholm , New J. Chem. 2020, 44, 20643–20650.

[28] H. D. Root , D. N. Mangel , J. T. Brewster , H. Zafar , A. Samia , G. Henkelman , J. L. Sessler , Chem. Commun. 2020, 56, 9994–9997. 10.1039/d0cc04400h 32724979

[29] J.-Y. Shin , T. Yamada , H. Yoshikawa , K. Awaga , H. Shinokubo , Angew. Chem. Int. Ed. 2014, 53, 3096–3101; 10.1002/anie.201310374 24554515

[30] J.-Y. Shin , Z. Zhang , K. Awaga , H. Shinokubo , Molecules 2019, 24, 2433. 10.3390/molecules24132433 PMC6651293 31269689

[31] N. C. Baird , J. Am. Chem. Soc. 1972, 94, 4941–4948.

[32] H. Ottosson , Nat. Chem. 2012, 4, 969–971. 23174974 10.1038/nchem.1518

[33] M. D. Peeks , J. Q. Gong , K. McLoughlin , T. Kobatake , R. Haver , L. M. Herz , H. L. Anderson , J. Phys. Chem. Lett. 2019, 10, 2017–2022. 30951313 10.1021/acs.jpclett.9b00623 PMC6488184

[34] P. B. Karadakov , M. Di , D. L. Cooper , J. Phys. Chem. A 2020, 124, 9611–9616. 33155798 10.1021/acs.jpca.0c08594

[35] D. Yu , C. Rong , T. Lu , P. Geerlings , F. De Proft , M. Alonso , S. Liu , Phys. Chem. Chem. Phys. 2020, 22, 4715–4730. 32057037 10.1039/c9cp06120g

[36] X. Jiang , S. D. Laffoon , D. Chen , S. Perez-Estrada , A. S. Danis , J. Rodrıguez-Lopez , M. A. Garcia-Garibay , J. Zhu , J. S. Moore , J. Am. Chem. Soc. 2020, 142, 6493–6498. 32208689 10.1021/jacs.0c01430

[37] R. Nozawa , H. Tanaka , W.-Y. Cha , Y. Hong , I. Hisaki , S. Shimizu , J.-Y. Shin , T. Kowalczyk , S. Irle , D. Kim , H. Shinokubo , Nat. Commun. 2016, 7, 13620. 27901014 10.1038/ncomms13620 PMC5141365

[38] R. Nozawa , J. Kim , J. Oh , A. Lamping , Y. Wang , S. Shimizu , I. Hisaki , T. Kowalczyk , H. Fliegl , D. Kim , H. Shinokubo , Nat. Commun. 2019, 10, 3576. 31395873 10.1038/s41467-019-11467-4 PMC6687811

[39] Y. Ni , T. Y. Gopalakrishna , H. Phan , T. Kim , T. S. Herng , Y. Han , T. Tao , J. Ding , D. Kim , J. Wu , Nat. Chem. 2020, 12, 242–248. 31959959 10.1038/s41557-019-0399-2

[40] J. Michl , Pure Appl. Chem. 2008, 80, 429–446.

[41] R. V. Williams , Chem. Rev. 2001, 101, 1185–1204. 11710217 10.1021/cr9903149

[42] P. B. Karadakov , D. L. Cooper , J. Phys. Chem. A 2016, 120, 8769–8779. 27739684 10.1021/acs.jpca.6b09426

[43] T. Stauch , Chem. Eur. J. 2018, 24, 7340–7344. 29575402 10.1002/chem.201801013

[44] N. Li , B. Wu , C. Yu , T. Li , W. X. Zhang , Z. Xi , Angew. Chem. Int. Ed. 2020, 59, 8868–8872; 10.1002/anie.201916651 32133711

[45] D. Geuenich , K. Hess , F. Köhler , R. Herges , Chem. Rev. 2005, 105, 3758–3772. 16218566 10.1021/cr0300901

[46] H. Fliegl , D. Sundholm , S. Taubert , J. Juselius , W. Klopper , J. Phys. Chem. A 2009, 113, 8668–8676. 19586004 10.1021/jp9029776

[47] H. Fliegl , S. Taubert , O. Lehtonen , D. Sundholm , Phys. Chem. Chem. Phys. 2011, 13, 20500–20518. 21909556 10.1039/c1cp21812c

[48] A. Ligabue , U. Pincelli , P. Lazzeretti , R. Zanasi , J. Am. Chem. Soc. 1999, 121, 5513–5518.

[49] G. Merino , T. Heine , G. Seifert , Chem. A Eur. J. 2004, 10, 4367–4371. 10.1002/chem.200400457 15352120

[50] P. Von Rague Schleyer , C. Maerker , A. Dransfeld , H. Jiao , N. J. R. van EikemaHommes , J. Am. Chem. Soc. 1996, 118, 6317–6318. 28872872 10.1021/ja960582d

[51] Z. Chen , C. S. Wannere , C. Corminboeuf , R. Puchta , P. von Rague Schleyer , Chem. Rev. 2005, 105, 3842–3888. 16218569 10.1021/cr030088+

[52] P. Von Rague Schleyer , M. Manoharan , Z. X. Wang , B. Kiran , H. Jiao , R. Puchta , N. J. Van Eikema Hommes , Org. Lett. 2001, 3, 2465–2468. 11483036

[53] S. Klod , E. Kleinpeter , J. Chem. Soc. Perkin Trans. 2 2001, 1893–1898.

[54] T. Heine , C. Corminboeuf , G. Seifert , Chem. Rev. 2005, 105, 3889–3910. 16218570 10.1021/cr030082k

[55] R. Gershoni-Poranne , A. Stanger , Chem. Eur. J. 2014, 20, 5673–5688. 24677667 10.1002/chem.201304307

[56] A. Stanger , G. Monaco , R. Zanasi , ChemPhysChem 2020, 21, 65–82. 31652016 10.1002/cphc.201900952

[57] B. J. Lampkin , P. B. Karadakov , B. VanVeller , Angew. Chem. Int. Ed. 2020, 59, 19275–19281; 10.1002/anie.202008362 33448542

[58] V. Vijay , M. Madhu , R. Ramakrishnan , A. Benny , M. Hariharan , Chem. Commun. 2019, 56, 225–228. 10.1039/c9cc07251a 31803867

[59] J. R. Cheeseman , J. Chem. Phys. 1996, 104, 5497–5509.

[60] J. C. Facelli , Prog. Nucl. Magn. Reson. Spectrosc. 2011, 58, 176–201. 21397119 10.1016/j.pnmrs.2010.10.003 PMC3058154

[61] T. Helgaker , S. Coriani , P. Jørgensen , K. Kristensen , J. Olsen , K. Ruud , Chem. Rev. 2012, 112, 543–631. 22236047 10.1021/cr2002239

[62] P. W. Atkins, Physical Chemistry, Oxford University Press, Oxford, 5th ed., **1994**.

[63] N. F. Ramsey , Phys. Rev. 1950, 78, 699–703.

[64] E. Steiner , P. W. Fowler , Chem. Commun. 2001, 1, 2220–2221. 10.1039/b104847n 12240120

[65] D. Sundholm , H. Fliegl , R. J. Berger , Wiley Interdiscip. Rev.: Comput. Mol. Sci. 2016, 6, 639–678.

[66] M. J. Frisch, G. W. Trucks, H. B. Schlegel, G. E. Scuseria, M. A. Robb, J. R. Cheeseman, G. Scalmani, V. Barone,B. Mennucci, G. A. Petersson, H. Nakatsuji, M. Caricato, X. Li, H. P. Hratchian, A. F. Izmaylov, J. Bloino, G. Zheng, J. L. Sonnenberg, M. Hada, M. Ehara, K. Toyota, R. Fukuda, J. Hasegawa, M. Ishida, T. Nakajima, Y. Honda, O. Kitao, H. Nakai, T. Vreven, J. A. Montgomery, Jr., J. E. Peralta, F. Ogliaro, M. Bearpark, J. J. Heyd, E. Brothers, K. N. Kudin, V. N. Staroverov, T. Keith, R. Kobayashi, J. Normand, K. Raghavachari, A. Rendell, J. C. Burant, S. S. Iyengar, J. Tomasi, M. Cossi, N. Rega, J. M. Millam, M. Klene, J. E. Knox, J. B. Cross, V. Bakken, C. Adamo, J. Jaramillo, R. Gomperts, R. E. Stratmann, O. Yazyev, A. J. Austin, R. Cammi, C. Pomelli, J. W. Ochterski, R. L. Martin, K. Morokuma, V. G. Zakrzewski, G. A. Voth, P. Salvador, J. J. Dannenberg, S. Dapprich, A. D. Daniels, O. Farkas, J. B. Foresman, J. V. Ortiz, J. Cioslowski, D. J. Fox, Gaussian09 Revision E.01, **2013**, Gaussian Inc. Wallingford CT.

[67] J. P. Perdew , K. Burke , M. Ernzerhof , Phys. Rev. Lett. 1996, 77, 3865–3868. 10062328 10.1103/PhysRevLett.77.3865

[68] C. Adamo , V. Barone , J. Chem. Phys. 1999, 110, 6158–6170.

[69] A. Schafer , H. Horn , R. Ahlrichs , J. Chem. Phys. 1992, 97, 2571–2577.

[70] F. Plasser , H. Lischka , J. Chem. Theory Comput. 2012, 8, 2777–2789. 26592119 10.1021/ct300307c

[71] F. Plasser , M. Wormit , A. Dreuw , J. Chem. Phys. 2014, 141, 024106. 25027998 10.1063/1.4885819

[72] F. Plasser , J. Chem. Phys. 2020, 152, 084108. 32113349 10.1063/1.5143076

[73] W. Humphrey , A. Dalke , K. Schulten , J. Mol. Graphics 1996, 14, 33–38. 10.1016/0263-7855(96)00018-5 8744570

[74] GIMIC version with support for cube files; available from https://github.com/felixplasser/gimic.

[75] Supporting research data available: Molecular geometries, Gaussian input/output files, input scripts for VMD. DOI: 10.17028/rd.lboro.13546826.

[76] C. Corminboeuf , T. Heine , G. Seifert , P. Von Rague Schleyer , J. Weber , Phys. Chem. Chem. Phys. 2004, 6, 273–276.

[77] A. Stanger , J. Org. Chem. 2006, 71, 883–893. 16438497 10.1021/jo051746o

[78] M. Sola , Front. Chem. 2013, 1, 4–11. 24790950 10.3389/fchem.2013.00022 PMC3982536

[79] J. P. Anhalt , E. W. Friend , E. H. White , J. Org. Chem. 1972, 37, 1015–1019.

[80] P. B. Karadakov , J. Phys. Chem. A 2008, 112, 7303–7309. 18636691 10.1021/jp8037335

[81] R. Ayub , O. E. Bakouri , K. Jorner , M. Sola , H. Ottosson , J. Org. Chem. 2017, 82, 6327–6340. 28535673 10.1021/acs.joc.7b00906

[82] S. Horn , F. Plasser , T. Müller , F. Libisch , J. Burgdörfer , H. Lischka , Theor. Chem. Acc. 2014, 133, 1511.

[83] H. Lischka , D. Nachtigallova , A. J. A. Aquino , P. Szalay , F. Plasser , F. B. C. Machado , M. Barbatti , Chem. Rev. 2018, 118, 7293–7361. 30040389 10.1021/acs.chemrev.8b00244

[84] S. Pathak , R. Bast , K. Ruud , J. Chem. Theory Comput. 2013, 9, 2189–2198. 26583713 10.1021/ct3011198

[85] L. J. Karas , C. H. Wu , H. Ottosson , J. I-Chia Wu , Chem. Sci. 2020, 11, 10071–10077. 34094268 10.1039/d0sc02294b PMC8162126

[86] R. Jasti , J. Bhattacharjee , J. B. Neaton , C. R. Bertozzi , J. Am. Chem. Soc. 2008, 130, 17646–17647. 19055403 10.1021/ja807126u PMC2709987

[87] S. E. Lewis , Chem. Soc. Rev. 2015, 44, 2221–2304. 25735813 10.1039/c4cs00366g

[88] B. M. Wong , J. Phys. Chem. C. 2009, 113, 21921–21927. 10.1021/jp9074674 PMC3317592 22481999

[89] D. Sundholm , S. Taubert , F. Pichierri , Phys. Chem. Chem. Phys. 2010, 12, 2751–2757. 20200754 10.1039/b922175a

[90] L. Stojanovic , S. G. Aziz , R. H. Hilal , F. Plasser , T. A. Niehaus , M. Barbatti , J. Chem. Theory Comput. 2017, 13, 5846–5860. 29140693 10.1021/acs.jctc.7b01000

[91] T. Iwamoto , Y. Watanabe , T. Sadahiro , T. Haino , S. Yamago , Angew. Chem. Int. Ed. 2011, 50, 8342–8344; 10.1002/anie.201102302 21770005

[92] E. J. Leonhardt , R. Jasti , Nat. Chem. Rev. 2019, 3, 672–686.

[93] T. Ito , Y. Hayashi , S. Shimizu , J.-Y. Shin , N. Kobayashi , H. Shinokubo , Angew. Chem. Int. Ed. 2012, 51, 8542–8545; 10.1002/anie.201204395 22811074

[94] S. Ukai , Y. H. Koo , N. Fukui , S. Seki , H. Shinokubo , Dalton Trans. 2020, 49, 14383–14387. 33047762 10.1039/d0dt03143g

[95] S. Van Damme , G. Acke , R. W. Havenith , P. Bultinck , Phys. Chem. Chem. Phys. 2016, 18, 11746–11755. 26762574 10.1039/c5cp07170d

[96] A. Stanger , Eur. J. Org. Chem. 2020, 3120–3127.

