# Supplementary material for: Visualisation of Chemical Shielding Tensors (VIST) to Elucidate Aromaticity and Antiaromaticity
Source: European J Org Chem. 2021 May 5;2021(17):2529–39. doi: 10.1002/ejoc.202100352 (PMC8251739; doi:10.1002/ejoc.202100352)
Supplement: Supplementary file 1 — Supplementary [file EJOC-2021-2529-s001.pdf]

European Journal of Organic Chemistry

Supporting Information

Visualisation of Chemical Shielding Tensors (VIST) to Elucidate Aromaticity and Antiaromaticity**

Felix Plasser* and Florian Glöcklhofer

Author Contributions

F.P. Conceptualization:Lead; Formal analysis:Lead; Methodology:Lead; Software:Lead; Writing – original draft:Lead; Writing – review & editing:Equal
F.G. Conceptualization:Supporting; Formal analysis:Supporting; Validation:Supporting; Writing – original draft:Supporting; Writing – review & editing:Equal

Contents

Section S1 - Further details on the VIST method 1

Section S2 - Benzene 2

Section S3 - Paracyclophanetetraene 2

Section S4 - Norcorrole dimer 4

Section S1 - Further details on the VIST method

The shielding tensor is in general represented by a non-symmetric matrix, which gives rise to two technical issues, which we shall discuss briefly in the following: (i) the presence of left eigenvectors and (ii) the occurrence of complex eigenvalues. In Eq. (6) of the main manuscript we have considered the right eigenvectors of the chemical shielding tensor and we will use these in the following unless noted otherwise. Alternatively, it would be possible to do the same decomposition using the left eigenvectors

$$\sum_{\gamma\in\{x,y,z\}} l_\gamma^{(i)}\sigma_{\gamma\beta} = l_\beta^{(i)} t^{(i)} \qquad i\in\{1,2,3\} \tag{S1}$$

This would yield the same eigenvalues $t^{(i)}$ as Eq. (6), considering that they are determined as the roots of the same characteristic polynomial, but it would generally produce different principal axes. Note that the two equations differ in the sense that the summation in Eq. (6) goes over the components of magnetic field B_β whereas the summation in Eq. (S1) is performed with respect to the formal nuclear moments μ_γ. It is not *a priori* clear which representation is better. In practice, we have found that the representations have a similar appearance only that the left eigenvectors are a bit tilted with respect to the right ones. To exemplify this, we show the right and left eigenvectors of the shielding tensors of anthracene in its singlet ground state in Fig. S1 (b,c) and (d,e), respectively. First, it is noted that the left eigenvalues are the same as the right eigenvalues meaning that the length of the axes and size of the spheres is the same. In the case of the NICS(0) tensors, we also find that, due to symmetry reasons the left eigenvectors are exactly the same as the right ones. When viewing the NICS(1) tensors we find that the left eigenvectors are somewhat tilted with respect to the molecular plane but that they, otherwise, have a similar appearance to the right eigenvectors. We found the same conclusion also for other examples: the left eigenvectors are similar to the right eigenvectors with the exception of being slightly tilted. We, thus, continue by viewing only the right eigenvectors and suggest using these for future applications.

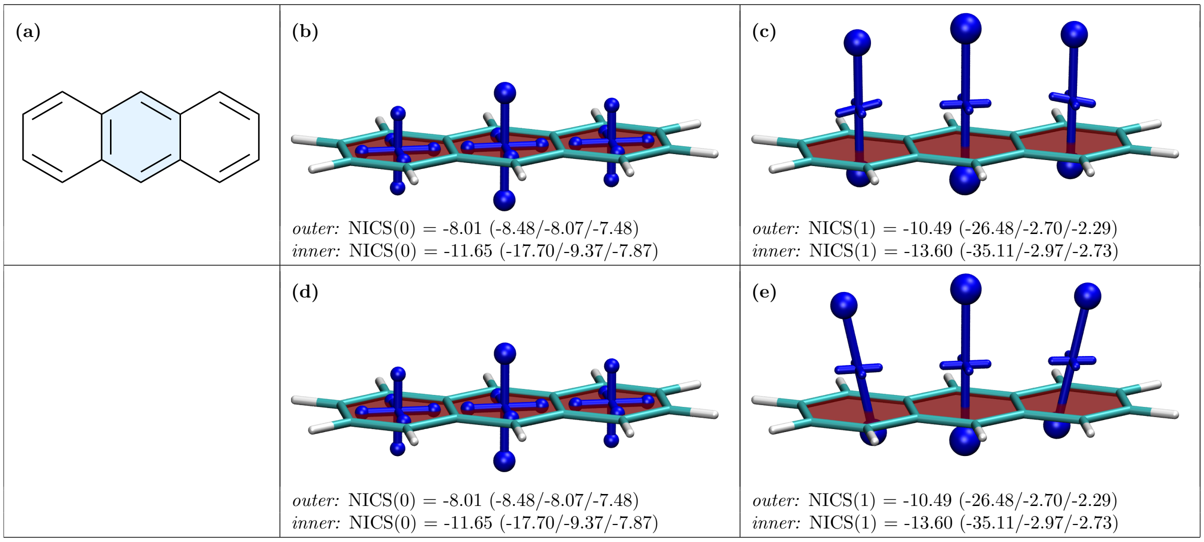

Figure S1: Analysis of local aromaticity in anthracene: (a) molecular structure with Clar sextet highlighted in blue; 3D representation of the chemical shielding tensors using the right eigenvectors computed at (b) the centre of each ring and (c) 1Å above the plane; (d) and (e) same as above using the left eigenvectors. NICS values (in ppm) are reported for the *outer* and *inner* rings.

Furthermore, we want to discuss the case where one of the eigenvalues, e.g. $t^{(1)}$, is a complex number. In this case, we use a mathematical formalism similar to the construction of two-dimensional real representations of cyclic symmetry groups. Due to the fact that the σ-matrix is real, it follows that if $t^{(1)}$ is complex, also its complex conjugate $t^{(1)*}$ is an eigenvalue and that the associated eigenvector is the complex conjugate of $\vec{q}^{(1)}$. In summary, we can write

$$t^{(2)} = t^{(1)*} \qquad \vec{q}^{(2)} = \vec{q}^{(1)*} \tag{S2}$$

Noting this relation, we use the following three vectors as a basis for the coordinate system: $\mathrm{Re}(\vec{q}^{(1)}), \mathrm{Im}(\vec{q}^{(1)}), \vec{q}^{(3)}$ where Re and Im denote the real and imaginary parts of the vectors. The associated diagonal matrix elements are $\mathrm{Re}(t^{(1)}), \mathrm{Re}(t^{(1)}), t^{(3)}$ noting that these conserve the trace in analogy to Eq. (8) of the main text, i.e.

$$\sigma_{\mathrm{iso}} = \frac{1}{3}\left(\mathrm{Re}(t^{(1)}) + \mathrm{Re}(t^{(1)}) + t^{(3)}\right) \tag{S3}$$

Graphical representation: In the VIST dumb-bell representation used, we consistently draw the length L of the axis and the radius R of the sphere as

$$L = 2 \times 0.3\sqrt{|t^{(i)}|} \qquad R = 0.03\sqrt{|t^{(i)}|} \tag{S4}$$

where $t^{(i)}$ is given in ppm and L and R are given in Å.

Section S2 - Benzene

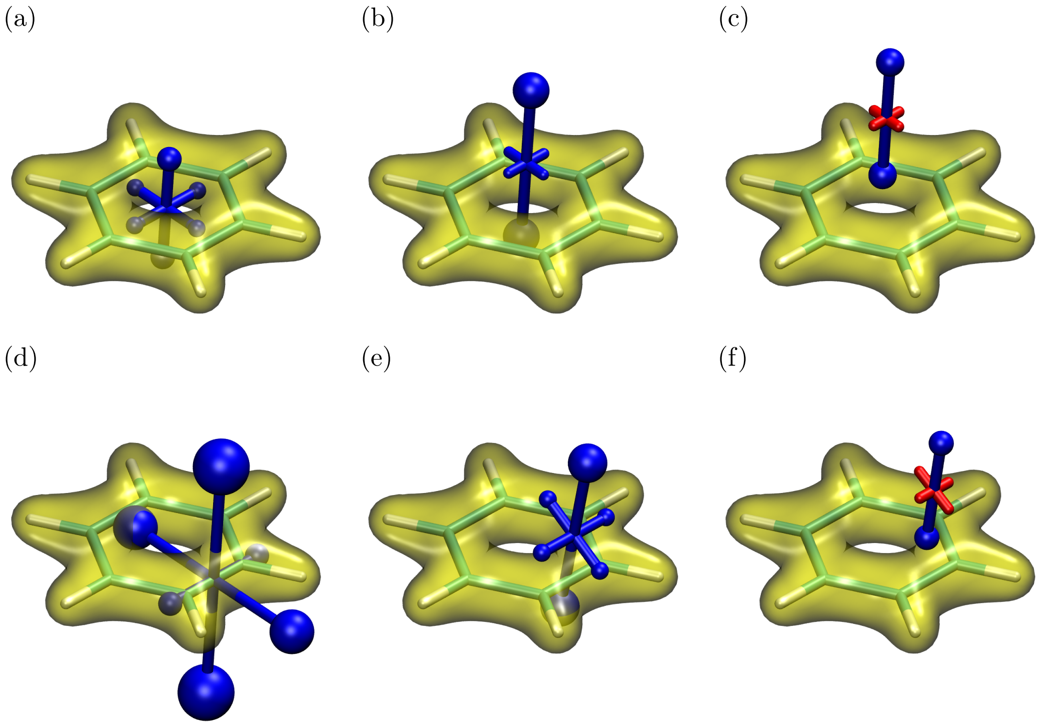

Figure S2: 3D visualisation of the chemical shielding tensors (VIST) in benzene. Negative (shielded/aromatic) contributions are shown in blue, positive (deshielded, antiaromatic) in red. Shielding tensors were computed at (a) the centre of the molecule, (b) 1 Å and (c) 2 Å above the plane; (d) at the centre of a bond, (e) 1 Å and (f) 2 Å above the bond. The electron density is shown in yellow (isovalue 0.1 a.u., encompassing about 60% of the total electron density).

Section S3 - Paracyclophanetetraene

Table S1: Geometric parameters for PCT in its neutral and dianion electronic states. Distances (d) are given in Å, dihedral angles (ϕ) in degrees; see the scheme below for atom numbering. The table indicates that upon formation of the dianion the formal single bond C_2-C_3 shortens and planarises whereas the double bond in the vinylene unit C_3=C_4 becomes longer and more twisted.

	$d(C_1{=}C_2)$	$d(C_2\text{-}C_3)$	$d(C_3{=}C_4)$	$\phi(C_1\text{-}C_2\text{-}C_3{=}C_4)$	$\phi(C_2\text{-}C_3{=}C_4\text{-}C_5)$
Neutral	1.408	1.466	1.351	28.6	8.0
Dianion	1.429	1.430	1.391	17.2	18.3

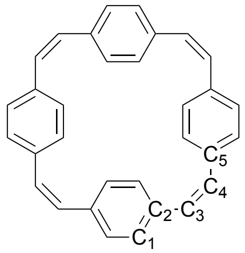

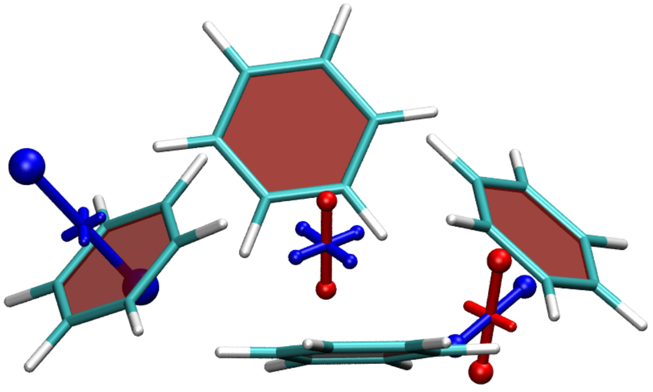

A: NICS(1) = -11.23 (-29.70/-2.26/-1.74)
B: NICS(0) = -0.31 (9.39/-5.16/-5.16)
C: NICS(0) = 1.31 (13.61/-12.18/2.49)

Figure S3: 3D visualisation of the chemical shielding tensors (VIST) of four isolated benzene rings arranged according to the geometry of PCT in its singlet ground state. This figure shows that deshielding at positions B and C is also present for isolated benzene rings. Comparison to Fig. 5 shows that deshielding is enhanced in the full macrocycle.

Section S4 - Norcorrole dimer

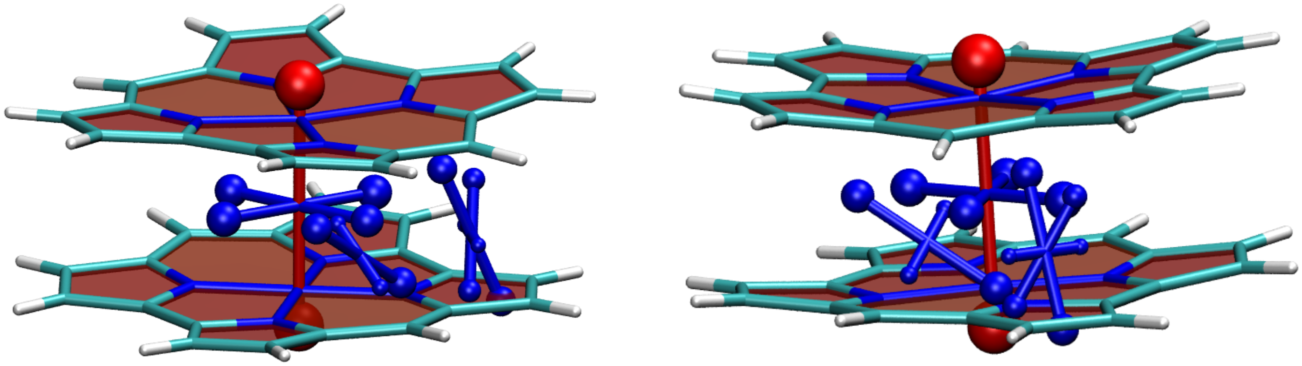

Figure S4: 3D visualisation of the chemical shielding tensors (VIST) in the norcorrole dimer using alternative viewing angles highlighting the tilt and non-orthogonality of the principal axes at the outer positions.
